# Diseases of the Nervous System

**Published:** 1906-03-17

**Authors:** 


### Progress in Medicine and Surgery.

### DISEASES OF THE NERVOUS SYSTEM.

(*Continued from page* 389.)

**Aphasia and Alexia.**—A considerable proportion of the cortex of the cerebrum appears to be devoted to the faculty of speech and the allied arts of reading and writing. For the exercise of these functions, as for other voluntary acts, there are required sensory impressions and motor impulses for the due reception and emission of which certain cerebral centres must be intact. These are the psychical centres of intelligent perception, the exact localisation of which in the hemispheres has not yet been determined; the sensory, receptive, or afferent centres; the motor, emissive, or efferent centres; and the association tracts, by which communication between these is made. The retention and recall of the various sensory impressions received from an object, those of sight, sound, touch, etc., constitute the mental image of that object; while the recollection of the sight or sound of its written or spoken name, and of the muscular actions concerned in speaking or writing the name, constitute the word-image. It is disturbance of the mechanism with which the formation of the word-image is concerned which constitutes the different forms of aphasia, alexia, and agraphia. In right-handed people these centres are situated on the left side of the brain. Of the sensory centres the *auditory speech centre*, that for the storing of the memory of the sound of spoken words, is situated in the posterior part of the first and second temporal convolutions; while the *visual speech centre*, that for the memory of the visual appearance of written and printed words, is found in the angular and inferior parietal convolutions. The *motor speech centre*, the well-known Broca's convolution, concerned with the motor memories of articulate speech, lies in the posterior part of the third frontal convolution. Association tracts of fibres are believed to connect these various centres with one another, and are by some thought to converge to an ideation centre in the Island of Reil. There are necessarily also connections with the general centre for vision in the occipital lobe, with that for hearing in the temporal lobe, and in the case of Broca's convolution with the motor cells in the medulla and cord, from which arise the nerves supplying the muscles concerned in articulation and writing, or in the upper neurone equivalents of these in the cerebrum. The existence of a separate independent centre for the memories of writing movements has been denied by some and affirmed by others. Some authorities suppose that the nervous impulses concerned in the production of written speech pass through the motor-vocal speech centre (Broca's centre) in order to reach the graphic or writing speech centre. Byrom Bramwell[7] is among those who for long have maintained that under normal circumstances the nervous impulses for written speech pass from the visual speech centre to the graphic centre directly, and not through the motor-vocal speech centre, and he cites a case recently observed by him in which pure motor aphasia was present without agraphia in support of this contention. The patient, a woman aged 27, who was convalescing from influenza, accompanied by sore throat, and later by phlebitis in the leg, was suddenly seized with cerebral symptoms, which resulted in right-sided hemiplegia, loss of speech, and pain in the left side of the head. There was some weakness on the left side, but it was more marked on the right, especially in the arm, which was weaker than the leg. Slight ankle clonus and an extensor response to plantar stimulation were obtained on this side. The right side of the face was paralysed, especially the lower muscles, but the orbicularis palpebrarum was also affected. There were also analgesia and anæsthesia on the right side of the face, impaired hearing in the right ear, impaired taste on the right side of the tongue, and loss of smell in the right nostril. The patient was right-handed. She understood everything that was said to her, and could read quite well, therefore there was no word deafness or word blindness. Neither was there hemianopsia, but she was totally unable to speak, therefore she had complete motor aphasia. She could, however, write, that is, no agraphia was present. Such writing disability as did appear was evidently due to difficulty in holding pencil or pen owing to partial paralysis of the right arm. The intellect appeared unaffected. No voluntary word was uttered, but by copying the movements of a speaker's lips the patient was able to whisper imperfectly a few words. By constant practice she learnt to repeat many sounds and words, and in three months' time could speak fairly well, but slowly, while under excitement speech became difficult and confused. Slight facial paralysis persisted. Mitral stenosis was present. The lesion was thought to be an embolism probably of the left middle cerebral artery or some of its branches. The sensory symptoms present, anæsthesia of the face and impairment of special senses, would point to a lesion of the posterior end of the internal capsule, but against this would be more marked paralysis of the arm and face than of the leg. Accepting the theory of embolism of the left middle cerebral artery, the lesion would appear to be a softening of the lower end of the motor area on the left side—a cortical and also a subcortical injury. The chief interest of the case lies in the existence of such complete motor-vocal aphasia without any agraphia, as the patient could write long letters when able only to laboriously pronounce a few syllables. A case of alexia has been reported by Edwin Bramwell[8] which was almost pure. The patient, a man aged sixty-three, suffered from chronic Bright's disease, with bronchitis and emphysema. He complained of his sight, and was found on examination to have good visual acuity, but to be the subject of right homonymous hemianopsia and word blindness. He was right handed, fairly intelligent, and showed no weakness of the right side. He understood everything that was said to him, so that his auditory speech centre was intact; he could also express himself fluently and well, so that his motor-vocal centres were unimpaired, but, with the exception of a very few single letters and monosyllabic words, he could not read written or printed matter, so that he had almost complete letter and word blindness. Numerals were, however, read fairly well. There was no agraphia, the patient wrote promptly and correctly anything he was asked to do, but the first letter of the sentence had sometimes to be written for him. He could not read what he had written longer than five minutes after doing so. It was thought that there was probably some slight defect of the visual word centre, but that the chief lesion, since the power of writing was practically retained, was occipital or subcortical, using the word subcortical in relation to the parietal cortex. Opportunity for a post-mortem verification of this diagnosis was given, as the patient died shortly with symptoms of uræmia. On examination a large area of softening was apparent in the posterior part of the left hemisphere, corresponding to the area supplied by the posterior cerebral artery. It was not of recent origin. The left occipital lobe was much involved, the cuneus being destroyed. There was also extensive softening of the under surface of the temporo-sphenoidal lobe, including the posterior two-thirds of the gyrus hippocampus, a large portion of the fourth, and the posterior portion of the third temporo-sphenoidal convolutions. The supramarginal convolution and the angular gyrus, except, perhaps, the lower portion of its posterior limb, were intact. This would account for the almost perfect retention of the power of writing, while the alexia was evidently due to destruction of the fibres connecting the left occipital lobe with the visual word centre. Several cases of word blindness have also been recorded by J. V. Paterson[9] who draws attention to the teaching of Bastian concerning the primary and fundamental importance, in normal individuals, of the auditory word centre in the production of speech. This and Broca's convolution have been termed by Wyllie the primary couple. The visual word centre and the writing centre are subsidiary, the artificial products of education. One of these patients combined alexia with nearly complete agraphia, right-sided hemiplegia, and hemianopsia. Intelligence was good and speech unimpaired. These lesions were probably due to a cortical softening over part of the area supplied by the left middle cerebral artery, including the visual word centre and the radiations of Gratiolet. Another patient, an intelligent old lady, complained of difficulty in reading, and was found to have right hemianopsia, with some difficulty in recognising words and in expressing herself in writing. Memory and intelligence were slightly failing. It was thought that thrombosis of the left posterior cerebral artery had damaged the occipital lobes and their connections with the visual word centre. A third patient also complained of difficulty in reading and inability to see objects on the right. The hemianopsia had apparently lasted four years. Attempts at reading soon produced exhaustion and inability to proceed, so that a dyslexia rather than an alexia was present, analogous to the cases of dyslexia described by Berlin, and probably due to an interruption in the conducting paths between the visual centres and the visual word centre.

[7] Lancet, Oct. 7. [8] Scottish Med. and Surg. Jour., July. [9] Ibid.

